# Preliminary report on a novel technique for endoscopic transaxillary thyroidectomy: a case–control study

**DOI:** 10.1097/JS9.0000000000000885

**Published:** 2023-11-16

**Authors:** Yang Liu, Jiazhong Wang, Shuo Chen, Hao Lv, Shuo Yu, Xiaoli Ran, Nan Gao, Yun Sun, Gang Cao

**Affiliations:** Department of General Surgery, Xi’an Jiaotong University Second Affiliated Hospital, Xi’an, People’s Republic of China

**Keywords:** endoscopy, thyroidectomy, transaxillary

## Abstract

**Background:**

Endoscopic transaxillary approaches to thyroidectomy have been well described and gasless transaxillary endoscopic thyroidectomy (GTET) is the most popular method. However, this require a single long axillary incision which is longer than most remote access thyroidectomy procedures. The authors improved the GTET and provided a novel way to access the thyroid. The purpose of this study was to test the feasibility of our novel transaxillary thyroidectomy procedure and to attempt to reduce the size of the scar and reduce the flap creation area.

**Methods:**

One hundred sixteen patients who underwent our novel transaxillary thyroidectomy procedure were compared with the patients who underwent open and GTET procedures. The patients’ demographics, outcomes, and complications were analyzed.

**Results:**

Although the operation time (121.48±23.91 mins) was longer in the novel endoscopic group compare to the open group, it was shorter than GTET group. Intraoperative blood loss was similar between the groups. However, the novel procedure group had more drainage volume within 48 postoperative hours compare to other two groups. Despite the VAS pain score did not reveal a difference between the open and novel endoscopic procedure, it was lower in the novel procedure than GTET. The hospital stay days did not show a difference between the two groups. The number of resected central lymph nodes was similar between the groups. Differences did not reveal between the groups regarding to the complications rate.

**Conclusion:**

Our results showed that our novel transaxillary thyroidectomy procedure is feasible and safe. This procedure can be an alternative endoscopic transaxillary method for thyroidectomy.

Highlights

- We provide a novel transaxillary thyroidectomy procedure.
- The novel transaxillary thyroidectomy procedure has advantages of shorter incision length and smaller flap creation area than conventional gasless transaxillary thyroidectomy procedure.
- The procedure was feasible and safe and did not increase the complications or change the outcome.

Remote thyroidectomy is popular with patients who have cosmetic requirements due to its nonscarring feature on the neck. There are several endoscopic thyroidectomy approaches, each with its own advantages and disadvantages^1,2^. Among them, transaxillary thyroidectomy is one of the most important surgical approaches.

Transaxillary thyroidectomy was first described by Ikeda in 2000^3^. They accessed the thyroid by dissecting the sternocleidomastoid muscle (SCM) off the sternohyoid with three ports in the axilla, then the SCM was pressed downward and the thyroid gland became visible. However, the SCM became an obstacle to surgical vision with Ikeda’s method, so a flexible endoscopy was used in his report^3^. In order to directly access the thyroid, Chung’s team improved the procedure and described a gasless transaxillary endoscopic thyroidectomy (GTET) procedure by lifting the clavicular head of SCM upward with a special retractor in 2006^4^. The GTET is currently the most widely used transaxillary thyroidectomy procedure in the world^2,5,6^. However, it requires a longer incision of 5–6 cm in the axilla, followed by the creation of a subcutaneous flap extending to the clavicle, so that a static elevating retractor could be placed^2,5,6^. Sometimes an additional 5 mm trocar is also required in the procedure. In contrast, other remote approaches like transoral and breast approaches just have several small trocar incisions^1,2^.

Our team improved Chung’s procedure in 2021 and named it Liu’s transaxillary thyroidectomy procedure. This procedure shortens the incision length and reduced the subcutaneous flap creation area. In this report, we share our preliminary experience with 116 cases using this novel procedure and compare them with the conventional open and GTET procedures.

## Method

### Patient selection

We collected clinical data retrospectively from July 2021 to May 2023, excluding the first 20 cases that underwent our novel transaxillary thyroidectomy, since we were not very proficient with the new technique and the standard protocol had not been established during the first 20 cases. Patients who underwent conventional open thyroid lobectomy by the same surgeon and all the GTET performed in our hospital during the same period were used as controls.

The Strengthening the Reporting of case–control study Studies in Surgery (STROCSS) guideline for observational studies was followed to protocol, conduct, and present this case–control study^7^. The study was registered in ClinicalTrials and approved by the Ethics Committee of Xi’an Jiaotong University, compliant with the Helsinki medical research ethical principles. The data are anonymous, and the requirement for informed consent was waived.

### Criteria of both conventional and novel endoscopic transaxillary thyroidectomy

The inclusion criteria for both GTET and our novel transaxillary thyroidectomy are as follows: (1) The patients with benign or malignant thyroid disease require lobe thyroidectomy; (2) Patients with cosmetic requirements who agreed to transaxillary thyroidectomy; (3) Benign lesions with the largest diameter is less than or equal to 5 cm; (4) Differentiated thyroid cancer (DTC) meets the following conditions simultaneously: ①The maximum diameter of the primary lesion is less than 2.5 cm; ②There is no extraglandular invasion or only minimal external invasion of the anterior thyroid capsule or minor invasion of the sternum thyroid muscle; Exclusion criteria: (1) the patients had poor compliance and could not be re-examined regularly; (2) Patients with a history of neck or thyroid surgery; (3) Patients requiring total thyroidectomy or lateral neck dissection. All the patients were informed of both open and transaxillary surgical methods and signed the consent form.

The open lobe thyroidectomies were done in patients who need lobe thyroidectomy treatment and refused or could not perform any kind of remote thyroidectomy procedures.

## Intervention

### Preoperative preparation

All the patients underwent routine blood tests, thyroid function tests, coagulation function, blood biochemistry, calcitonin, carcinoembryonic antigen, neck and chest CT, neck ultrasound, electronic laryngoscopy, and other examinations. The patients were not allowed to eat or drink 12 h before surgery.

## Transaxillary thyroidectomy surgical procedure

**Figure 1:**
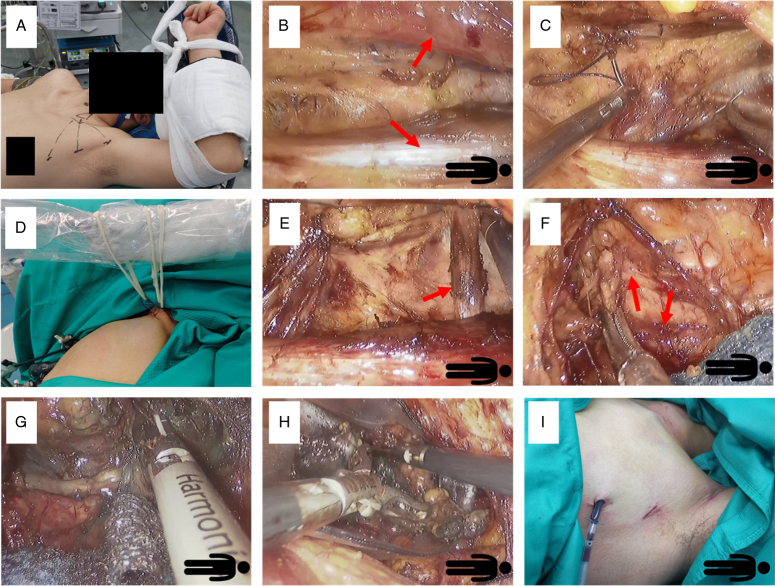
The key steps of the novel transaxillary thyroidectomy procedure. A: shows the body position and incision site. B: central neck region was accessed between the heads of SCM (arrow shows the clavicular and lateral head of SCM). C–E: the clavicular head of SCM and trap muscle was pulled upward by several percutaneous sutures and the suture was fixed on the anesthesia frame by rubber band traction (arrow shows the omohyoid muscle). F–G: Expose the RLN and dissociate the thyroid lobe (arrow shows the RLN). H: the specimen was taken out by plastic bag. I: shows the incision site.

The patient was placed in a supine neck hyperextension position while the upper limb of the diseased side was in a salute position. Two 5 mm incisions were made at the top of the axillary crease and the apex of the outer upper quadrant of the mammary gland as an operating port. A 10 mm incision was made at the midpoint of the line connecting the two points as an observation port (Fig. 1A). Trocars were inserted and a subcutaneous flap was created with a CO2 inflation pressure of 6 mmHg. The lower border of the flap was the collarbone, the up border was the clavicular head of SCM, and the lateral border was the connection line between the middle point of the clavicular head of SCM and the collarbone. The SCM was dissociated between its sternal and clavicular head (Fig. 1B). Then the sternocleidomastoid clavicular head was pulled upward via transcutaneous sutures and the sutures were pulled with a ribbon hanging on the anesthesia frame. The omohyoid muscle was freed, then the internal jugular vein and common carotid artery were carefully exposed and protected. The strap muscles together with the thyroid lobe were lifted with pull sutures through the skin again (Fig. 1C-E). The lower parathyroid glands together with their vasculature were identified and preserved in situ. If the lower parathyroid could not be preserved, try to find it in the surgical specimens and transplant it in SCM. The recurrent laryngeal nerve and the upper parathyroid glands were identified and kept in situ (Fig. 1F, G). The vessels around the thyroid were dissociated and the affected side thyroid lobe was removed (Fig. 1H). If needed, the central neck lymph nodes on the same side as the patient’s thyroid lobe were removed. A drainage tube was placed postoperation and then removed when the drainage volume was less than 15 ml/day.

The conventional open thyroidectomies and GTET were performed according to guidelines for the surgical management of thyroid disease and Chung’s description^6,8,9^.

### Comparison and outcomes

The study analyzed various variables to compare surgical outcomes among patients undergoing open thyroidectomy, GTET, and our novel procedure. These variables included total operation time, intraoperative bleeding volume, number of removed lymph nodes, drainage volume during the first 48 h, duration of drainage, VAS score after 24 h of surgery, hospital stay duration, presence of parathyroid during pathology examination, and postoperative complications and complications.

### Statistics

The continuous data were presented as the mean±SD, and the Kolmogorov–Smirnov test was applied to ensure normal distribution. The *t*-test was used to analyze differences between groups if the data were normally distributed, while the Mann–Whitney *U* test was used if the data did not conform. Categorical variables were presented as numbers, and differences between groups were tested using a χ^2^ test. The data were analyzed using the reliable Prism 8.0 software. A *P*<0.05 is considered significant.

## Result

A total of 116 patients who underwent our novel procedure, 42 patients who underwent GTET and 110 open thyroidectomies were included in the study. The characteristics of the patients are shown in Table 1. The results showed that more women underwent the novel transaxilllary procedure than the open (78.45 VS. 65.45%, *P*<0.05), and the age in the novel transaxillary group was younger than that in the open group (39.86 VS. 52.15 years, *P*<0.05). However, there was no significant difference in age and sex between the novel procedure and GTET groups. The other characteristics, such as BMI, DTC proportion, and lesion size, did not show any significant difference between the groups (Table 1).

**Table 1:** Features of patients between the groups.

				*P*	
	Open (*n*=110)	GTET (*n*=42)	Laparoscopy (*n*=116)	Novel vs. open	Novel vs. GTET
Male	38	10	25	0.04*	0.93
Female	72	32	91		
Age (years)	52.15±12.32	41.61±8.52	39.86±12.67	<0.01*	0.41
BMI	24.05±3.22	23.72±3.43	24.18±3.71	0.78	0.54
Left	58	20	56	0.59	0.91
Right	52	22	60		
Lobectomy	59	25	63	0.97	0.31
Lobectomy + CND	51	17	53		
Merge Hashimoto	16	6	14	0.72	0.92
Benign diseases	45	15	30	0.02*	0.31
DTC	65	27	86		
Cyst size (mm)	33.42±17.28	32.25±15.34	31.85±12.41	0.15	0.87
Tumor size (mm)	10.29±6.48	12.51±6.82	11.72±8.24	0.43	0.58

All the transaxillary operations were performed successfully without conversion to open surgery. However, one patient who underwent the novel procedure experienced a 5 mm rupture of the internal jugular vein during the operation, which was fixed with a 5-0 Prolene suture under endoscopy. Among the novel transaxillary procedure, 63 cases were lobectomy and 53 cases were combined with central neck dissection (CND), which the operation distribution was similar to conventional open operation and GTET. The operation time of novel transaxillary procedure was 121.48±23.91 mins which was longer than that of conventional open surgery but shorter than that of GTET (*P*<0.05). The intraoperative blood loss was the same among the procedures, and the bleeding volume was similar between lobectomy and combine CND in novel transaxillary procedure. In patients who underwent combined CND, an average of 7.05±2.98 lymph nodes were resected, which did not show a significant difference from conventional open surgery (7.51±4.53, *P*>0.05) and GTET (6.90±3.58, *P*>0.05)procedure.

In our novel transaxillary thyroidectomy patients, the drainage volume within the first postoperative 48 h was 80.40±69.09 ml, and the drainage tube was removed at 3.98±1.39 postoperative days. The patients were discharged at 2.12±0.89 days after the novel transaxillary procedure which was similar to the other two groups (*P*>0.05). The VAS pain score was similar between the novel and open procedures (*P*>0.05), but it was lower in novel procedure group than GTET after 24 h postoperatively (*P*<0.05). The transaxillary procedure showed a more drainage volume and drainage stay time than the open procedure (*P*<0.05), but the hospital stay time was similar between the procedures. The parathyroid gland was revealed in 19 cases during pathologic examination in the novel transaxillary procedure which did not show a significant difference from conventional open surgery and GTET (Table 2).

**Table 2:** Outcomes of patients between groups.

				P	
	Open (*n*=110)	GTET (*n*=42)	Laparoscopy (*n*=1160)	Novel vs. open	Novel vs. GTET
Operation time (min)	95.43±27.8	139.42±35.8	121.48±23.91	<0.01*	<0.01*
bleeding (ml)	16.54±23.19	21.64±19.67	22.41±22.76	0.21	0.86
Lymph nodes resected	7.51±4.53 (*n*=51)	6.90±3.58 (*n*=20)	7.05±2.98 (*n*=53)	0.36	0.79
Drainage during first 48 h (ml)	48.57±35.01	51.24±51.12	80.40±69.09	<0.01*	0.01*
Drainage stays (days)	3.65±0.97	3.51±1.58	3.98±1.39	0.04*	0.07
VAS	2.12±1.69	2.33±1.55	1.83±1.64	0.10	0.04*
Hospital stays (days)	2.29±0.73	2.24±0.87	2.12±0.89	0.12	0.46
Parathyroid was found during pathology examination	10	6	19	0.16	0.94

The 30-days postoperative complication rate was 7.27% (*n*=8) in the conventional surgery, 11.90% (*n*=5) in GTET, and 9.48% (*n*=11) in the novel endoscopy group. Two patients in the novel endoscopy group developed a postoperative hematoma at the top trocar tunnel and skin flap, which were successfully managed by applying a compressive dressing without re-exploration. Vocal cord paralysis was present in seven patients. Unfortunately, one of these patients was diagnosed with permanent recurrent laryngeal nerve injury by laryngoscopy. Another patient who underwent the novel procedure showed voice change right after the operation and was diagnosed with arytenoid subluxation by laryngoscopy. The patient’s voice fully recovered after surgical relocation. There were no other complications during the short-term follow-up, such as infection, lymphatic fistula, tracheal or esophageal leakage, etc. in novel operation. All the outcomes did not show a significant difference among the groups (*P*>0.05, Table 3), and all the patients did not develop recurrence during short-term follow-up.

**Table 3:** Comparison of complications between the groups.

				P	
	Open (*n*=110)	GTET (*n*=42)	Laparoscopy (*n*=116)	Novel vs. open	Novel vs. GTET
Total complications	8	5	11	0.55	0.66
Temporary laryngeal nerve palsy	6	3	7	0.81	0.80
Permanent RLN injury	0	1	1+9	0.33	0.45
Hematoma	1	1	2	0.59	0.79
Other	1 (infection)	0	1 (arytenoid subluxation)	0.97	0.55

## Discussion

In this study, we introduced a novel transaxillary thyroidectomy procedure that offers smaller incisions as its most promising advantage (Fig. 1 I). Although, the initial transaxillary procedure is completed with three ports, few surgeons use this method because the SCM could not be effectively pulled. With this method, the thyroid was reached between SCM and trap muscle approach, and SCM blocks the surgical working space and makes lobectomy difficult to complete, especially in the case which also requires CND. Therefore, the most popular transaxillary thyroidectomy method currently is GTET with an intra-SCM approach. However, GTET requires a longer incision of about 5–6 cm to insert an elevating retractor to lift the SCM. Sometimes, a 5 mm trocar incision is also needed. The core step of our new procedure is that we lifted SCM, and sternohyoid together with the thyroid lobe by suture instead of the retractor. The suture was pulled by a ribbon so the retraction strength could keep in a proper range. The suture was removed right after the operation and no scar was found at the suture site after the operation. Therefore, thyroidectomy could easily be completed with 3-ports of a total 2 cm incision length from the axilla (Fig. 1 I). In addition, our incisions were made in the skin folds of the axilla. So after several months, the scars were well hidden in the axillary skin folds and get an exceptional cosmetic effect.

**Figure 2:**
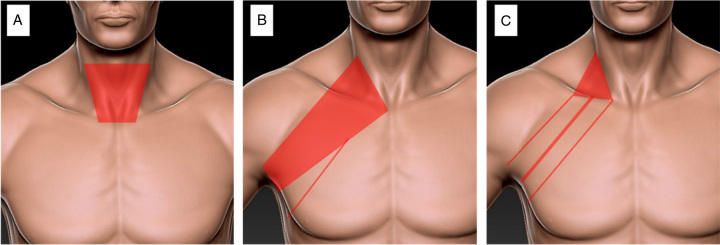
Comparison the flap creation area between different procedures. A: conventional open thyroidectomy; B: gasless transaxillary endoscopic thyroidectomy (GTET); C: our novel transaxillary thyroidectomy gas inflation endoscopic thyroidectomy.

Another advantage of this method is that less subcutaneous flap creation area is required. According to our calculation, the subcutaneous flap area of our operation only accounts for 27% [Using ImageJ software (version 1.53k; National Institutes of Health, Bethesda)] of conventional gasless transaxillary thyroidectomy and 56% of traditional open thyroidectomy (Fig. 2 A-C). Therefore, the average operation time of our novel procedure is much less than the operation time of GTET, although it is still more than the open procedure in the study. On the other hand, a smaller flap creation area is considered to reduce sensory loss or postoperative pain after thyroidectomy^10^. Although some studies revealed that conventional GTET had a higher postoperative pain score than open procedure^11^, no difference was revealed in the study between our novel procedure and the open procedure regarding to the 24 postoperative hours VAS score. The VAS pain score in our novel procedure was lower than the score in conventional GTET group. What is interesting is that our new procedure had a smaller dissection area, the drainage volume was more than the GTET and open procedure. This was probably because saline was injected subcutaneously to separate the flap in the transaxillary procedure but not in the GTET and open procedure.

Gas inflation is another prominent difference between our procedure and GTET. Although it is reported that inflation can cause subcutaneous emphysema, mediastinal emphysema, elevated PaCO2, and severe tachycardia^12^, it is also reported that moderate carbon monoxide stress will not lead to these adverse physiological effects. The incidence of CO2 embolism in transoral thyroidectomy which is mostly performed with gas inflation is very low, and only a few cases of CO2 embolism have been reported in the literature as a case report, and most of these cases were caused by large venous bleeding or high pneumoperitoneum pressure^13,14^. According to the studies from transoral surgery, when CO2 pressure kept at 4–6 mmHg, and CO2 flow rate was lower than 8–10 l/min, it would effectively reduce the occurrence of these inflation related complications. Although it is reported that 16.7% of patients had hypercapnia, serious, and persistent effects on patients due to hypercapnia is very rare during both transoral and breast approach thyroidectomy^15^. In our procedure, the CO2 pressure and flow rate were set 6 mmHg and 10 l/min based on the experience form transoral procedure, and none of patients had severe CO2 inflation related complications. In fact, in this surgical method, we only need to use inflation to maintain the operating space during the process of flap creating. After percutaneous suspension, CO2 will not be necessary for operation space maintaining. At this circumstance, the only purpose of CO2 kept inflation was to expel the smoke which produces during the operation and kept a clean surgical field vision.

In fact, our procedure is a modified method based on GTET, and technically same as GTET except for what we mentioned above. This study critically analyzes the safety and efficacy of our novel transaxillary thyroidectomy procedure. The result showed that we had the same resected lymph nodes number, bleeding volume with open procedure, and most importantly, this operation did not increase the complications incidence from the study. However, this study also has some limitations. Because the young aged and female patients are more likely to choose remote thyroidectomy when it is feasible^16^, therefore, the age and sex were different between the open and endoscopic groups in the study. Also, the proportion of benign disease is higher in open group since a larger cyst is a challenge for transaxillary thyroid lobectomy and most of DTC patients were with T1 in size. So, the difference in some features of the patients when compared the endoscopy groups and open group was one of limitations of the study. Even though, the results could initially show that our novel transaxillary thyroidectomy is feasible and safe.

In conclusion, we provide a novel surgical procedure of transaxillary thyroidectomy, which has a short incision length and smaller flap creation area. Although this method prolonged the operation time and increased drainage volume compared with conventional open surgery, it is safe and feasible. Additional, this procedure can be an alternative endoscopic transaxillary method for thyroidectomy only after a multicenter assessment.

## Ethical approval

This study was approved by the Ethics Committee of Second Affiliated Hospital of the Xi’an Jiaotong University (2023374).

## Consent

The data are anonymous, and the requirement for informed consent was therefore waived.

## Sources of funding

No funding.

## Author contribution

Y.L.: designed the procedure, conducted procedure, did the operations, and wrote the manuscript; S.C., J.W., S.Y., and H. L.: conducted procedure and acquired data; X.R., N.G., Y.S.: nursed the patients and followed up the patients; G.C.: conducted procedure, did the operations, and revised the manuscript. All authors revised and approved the manuscript for publication.

## Conflicts of interest disclosure

The authors declare no competing interests.

## Research registration unique identifying number (UIN)

The research has registered at ClinicalTrials(NCT05735054). Hyperlink to your specific registration: https://clinicaltrials.gov/ct2/show/study/NCT05735054?term=NCT05735054&draw=2&rank=1.

## Guarantor

Yang Liu.

## Data availability statement

The datasets used or analyzed during the current report are available from the corresponding author on reasonable request.

## Provenance and peer review

Not commissioned, externally peer-reviewed.
